# Changes in Incidence and Epidemiological Characteristics of Pulmonary Tuberculosis in Mainland China, 2005-2016

**DOI:** 10.1001/jamanetworkopen.2021.5302

**Published:** 2021-04-09

**Authors:** Hui Jiang, Mengyang Liu, Yingjie Zhang, Jinfeng Yin, Zhiwei Li, Chendi Zhu, Qihuan Li, Xiangyu Luo, Tingting Ji, Junjie Zhang, Yang Yang, Xiaonan Wang, Yanxia Luo, Lixin Tao, Feng Zhang, Xiangtong Liu, Weimin Li, Xiuhua Guo

**Affiliations:** 1 Beijing Chest Hospital, Capital Medical University, Beijing, China; 2 Beijing Tuberculosis and Thoracic Tumor Research Institute, Beijing, China; 3 School of Public Health, Capital Medical University, Beijing, China; 4 Beijing Municipal Key Laboratory of Clinical Epidemiology, School of Public Health, Capital Medical University, Beijing, China; 5 Chinese Center for Disease Control and Prevention, Beijing, China; 6 National Tuberculosis Clinical Lab of China, Beijing Tuberculosis and Thoracic Tumour Research Institute and Beijing Key Laboratory in Drug Resistance Tuberculosis Research, Beijing, China; 7 Institute of Statistics and Big Data, Renmin University of China, Beijing, China; 8 School of Life Sciences, Beijing Normal University, Beijing, China; 9 Department of Biostatistics, University of Florida, Gainesville

## Abstract

**Importance:**

The World Health Organization End TB (Tuberculosis) Strategy aims to decrease the global incidence and mortality of TB by 90% and 95%, respectively, as of 2035.

**Objective:**

To characterize the recent epidemiological trend of pulmonary TB (PTB) in mainland China based on the national surveillance data.

**Design, Setting, and Participants:**

This cross-sectional study collected demographic and clinical data of all patients reported in the national Tuberculosis Information Management System of China from January 1, 2005, to November 21, 2016. Data were analyzed from December 1, 2019, to July 31, 2020.

**Exposures:**

Pulmonary TB was defined as bacteriologically confirmed or clinically diagnosed TB in the lung parenchyma or the tracheobronchial tree.

**Main Outcomes and Measures:**

Temporal and spatial variation of annual incidence and demographic features of PTB in mainland China.

**Results:**

In total, 10 582 903 patients with PTB were reported in mainland China from 2005 to 2016. The median age of patients with PTB was 46 (interquartile range [IQR], 30-61) years, and 28.53% were 60 years or older. Most patients with PTB were male (69.8%) and farmers or herders (70.0%). The mean (SD) incidence of PTB was 66.61 (8.09) per 100 000 population. The annual incidence decreased from 72.95 per 100 000 population in 2005 to 52.18 per 100 000 population in 2016, and the reduction was greater in the eastern and central regions (31.6%; from 69.43 to 47.48 per 100 000 population) than in the western region (21.0%; from 82.06 to 64.82 per 100 000 population). Xinjiang Uygur Autonomous Region (135.03 per 100 000 population), Guizhou Province (115.98 per 100 000 population), and the Tibet Autonomous Region (101.98 per 100 000 population) had the highest mean annual incidences. The median time from onset of illness to diagnosis decreased from 36 (IQR, 16-92) days from 2005 to 2007 to 31 (IQR, 15-63) days in 2008 and later (*P* < .001) and was longer in the western region than in the eastern and central regions (41 [IQR, 20-91] vs 30 [IQR, 13-61] days; *P* < .001).

**Conclusions and Relevance:**

Although this study found that the incidence of PTB in mainland China showed a downward trend from 2005 to 2016, to achieve the World Health Organization 2035 goal, innovative and more efficient prevention and control strategies are needed, particularly among the most susceptible population, that is, farmers and herders in western China.

## Key Points

**Question:**

Have the incidence and epidemiological characteristics of pulmonary tuberculosis (PTB) changed in China from 2005 to 2016?

**Findings:**

In this cross-sectional study of the Chinese population, a total of 10 582 903 patients with PTB were reported from 2005 to 2016. The annual incidence ranged from 72.95 per 100 000 population in 2005 to 52.18 per 100 000 population in 2016, with a mean incidence of 66.61 per 100 000 population; the median patient age was 46 years, and 70.1% were farmers and herders.

**Meaning:**

These findings suggest that preventive measures for PTB should be based on the results of epidemiological investigation.

## Teaser

This cross-sectional study assesses the recent epidemiological trend of pulmonary tuberculosis in mainland China based on national surveillance data.

## Introduction

Tuberculosis (TB), an infectious disease caused by *Mycobacterium tuberculosis*, is not only among the top 10 causes of death in the world but also the leading fatal single infectious disease.^1^ Three nationwide surveys regarding TB epidemiology in mainland China showed a decline in the prevalence of TB by more than 50% during the past 20 years owing to the broad adoption of a directly observed treatment, short-course strategy (DOTS) and the End TB Strategy.^2,3^ The annual TB incidence and mortality in China decreased by a mean of 3.2% and 7.7%,^4^ respectively, which is higher than the world mean (2.0% and 3.0%, respectively).^5^ Nevertheless, the number of new cases in China in 2017 was the second highest in the world despite the ongoing interventions.

After the severe acute respiratory syndrome outbreak in 2003, China established its first web-based National Notifiable Infectious Disease Surveillance System for 39 reportable infectious diseases,^6^ including TB. To collect more comprehensive information about patients with TB, starting January 1, 2005, China developed an additional web-based national TB surveillance system, the Tuberculosis Information Management System (TBIMS), to which all TB health facilities are required to report diagnosed cases of TB.^7^ The TBIMS allows real-time monitoring of TB diagnosis, treatment, and outcomes in China, especially for pulmonary TB (PTB).

In this study, we collected PTB data reported to the TBIMS from January 1, 2005, to November 21, 2016, and characterized the epidemiology of PTB in mainland China, focusing on changes in annual incidence, demographic characteristics, geographic patterns, seasonal patterns, and delay in diagnosis. To our knowledge, this is the largest retrospective epidemiological study on PTB based on the TBIMS in China.

## Methods

### Ethical Approval

Data from patients with TB were reported to the TBIMS as part of routine public health surveillance, and no informed consent was required according to the National Health Commission of the People’s Republic of China. The ethics committee at the Beijing Chest Hospital concluded that this cross-sectional study was exempt from institutional review because only deidentified data were analyzed. The methods and findings from this study are reported in accordance with the Strengthening the Reporting of Observational Studies in Epidemiology (STROBE) reporting guideline.

### Case Definition

We defined PTB as bacteriologically confirmed or clinically diagnosed TB in the lung parenchyma or the tracheobronchial tree. Because of lesions in the lungs, miliary TB was considered PTB. A patient with both pulmonary and extrapulmonary TB was classified as having PTB.^8,9^

Bacteriological diagnosis was based on test results of sputum smear or isolated culture as the reference standard. Clinical diagnosis was based on chest imaging (radiography or computed tomography), supplemented by epidemiological investigation, clinical manifestation (coughing, expectoration ≥2 weeks, or hemoptysis), or results of an immunology test (tuberculin skin test and/or interferon gamma release assay).^10^

### Data Sources

From January 1, 2005, the Ministry of Health of China launched the TBIMS, covering all TB control institutions (health care centers dedicated to TB prevention, treatment, and research).^11^ This system collects demographic, diagnosis, management, and outcome data about each patient with TB. In this study, we extracted from TBIMS demographic information (sex, age, occupation, ethnicity, and residence province), illness onset date, diagnosis date, and clinical outcomes (radiography, sputum smear results, and sputum culture results) of patients with PTB from January 1, 2005, to November 21, 2016. Data analysis was conducted from December 1, 2019, through July 31, 2020, and the data from 2005 to 2016 are the longest and most recently available we could obtain. Meanwhile, we obtained annual population statistics in different provinces from the National Bureau of Statistics of the People’s Republic of China to calculate the PTB incidence.^12^

### Statistical Analysis

We divided the 31 provinces in mainland China into 3 regions: western, central, and eastern. We calculated the overall and provincial PTB annual incidence (per 100 000 population) by testing method and age group. Because the data for December 2016 were not available, we estimated the number of cases in December using the mean number of cases in the first 11 months to calculate the incidence of 2016. To quantify seasonal patterns of PTB by province, we used a heat map of proportions of weekly case numbers among the annual total case number, with the means calculated during the study years. A joinpoint regression of annual incidences over time was used to identify change points in the temporal trend of PTB incidence. We stratified subsequent analyses by the periods defined by the change points. A ring map was made using ArcGIS, version 10.4 (Environmental Systems Research Institute, Inc) to demonstrate the spatiotemporal pattern of PTB incidence at the provincial and annual levels.

We fitted parametric (Weibull, gamma, and log-normal) and nonparametric (kernel density) distributions to the time from illness onset to diagnosis. Model fitness was visually examined by comparing a fitted density curve to the observed frequencies, and, when necessary, parametric distributions were compared using the Akaike information criterion.^13^ This analysis of diagnostic delay was performed by study period, age group, region, sex, and occupation.

We used SAS, version 9.4 (SAS Institute Inc) and R, version 3.6.0 (R Project for Statistical Computing) for data management and analysis. All statistical tests were 2-sided with a significance level of *P* < .05.

## Results

### Demographic Characteristics

A total of 10 582 903 patients with confirmed PTB were reported to the TBIMS from January 2005 to November 2016, with the highest number recorded in 2007 (n = 1 010 896). The age distribution remained basically unchanged over time, with a median of 46 (interquartile range [IQR], 30-61) years; 28.53% were 60 years or older, and 0.8% were younger than 15 years (Table 1 and eFigure 1A in the Supplement). Most patients (69.8%) were male compared with 30.2% female, and male patients tended to be older (69.9% vs 30.1% were 15 years or older) (Table 1 and eFigure 1B in the Supplement). Difference in sex was less prominent in pediatric patients (aged <15 years), with 52.7% male and 47.3% female. The age distribution was not significantly different across time periods and regions (eFigure 1C and 1D in the Supplement). Overall, patients with PTB were evenly distributed across regions, but pediatric patients were mostly found in the west (50.8%). Regarding occupation, farmers and herders accounted for 70.0% of PTB diagnoses. Patients with PTB were dominantly the Han ethnic group (92.0%), followed by Uighur (1.9%) and Zhuang (1.2%) (eTable 1 in the Supplement).

**Table 1.** Frequency of Patients With PTB Infection by Demographic Characteristics in Mainland China, January 2005 to November 2016

Characteristic	Patient group^a^		
	All (n = 10 582 903)	Aged <15 y (n = 81 587)	Aged ≥15 y (n = 10 500 750)
Sex			
Male	7 383 723 (69.8)	43 035 (52.7)	7 340 621 (69.9)
Female	3 198 703 (30.2)	38 552 (47.3)	3 160 129 (30.1)
Unknown	477 (0.005)	0	0
Age, median (IQR), y	46 (30-61)	12 (8-14)	47 (30-62)
Age group, y			
0-14	81 587 (0.8)	81 587 (100)	NA
15-29	2 524 106 (23.9)	NA	2 524 106 (24.0)
30-44	2 359 285 (22.3)	NA	2 359 285 (22.5)
45-59	2 597 934 (24.5)	NA	2 597 934 (24.7)
≥60	3 019 425 (28.5)	NA	3 019 425 (28.8)
Unknown	566 (0.005)	NA	0
Region			
Eastern	3 324 754 (31.4)	14 239 (17.5)	3 310 510 (31.5)
Central	3 813 220 (36.0)	25 925 (31.8)	3 787 242 (36.1)
Western	3 443 835 (32.5)	41 422 (50.8)	3 402 382 (32.4)
Unknown	1094 (0.01)	1 (0.001)	616 (0.006)
Occupation			
Nursery children	3361 (0.03)	3361 (4.1)	NA
Stay-home children	6674 (0.1)	6674 (8.2)	NA
Students	485 367 (4.6)	58 191 (71.3)	427 168 (4.1)
Farmers and herders	7 403 689 (70.0)	NA	7 403 689 (70.5)
Commercial service stratum	969 490 (9.2)	NA	969 490 (9.2)
Workers	563 010 (5.3)	NA	563 010 (5.4)
Other	1 151 312 (10.9)	13 361 (16.4)	1 137 393 (10.8)

Abbreviations: IQR, interquartile range; NA, not applicable; PTB, pulmonary tuberculosis.

^a^ Unless otherwise indicated, data are expressed as number (percentage) of patients. Percentages have been rounded and may not total 100. A total of 566 individuals did not give age data.

### Incidence and Epidemic Characteristics

**Figure 1.**
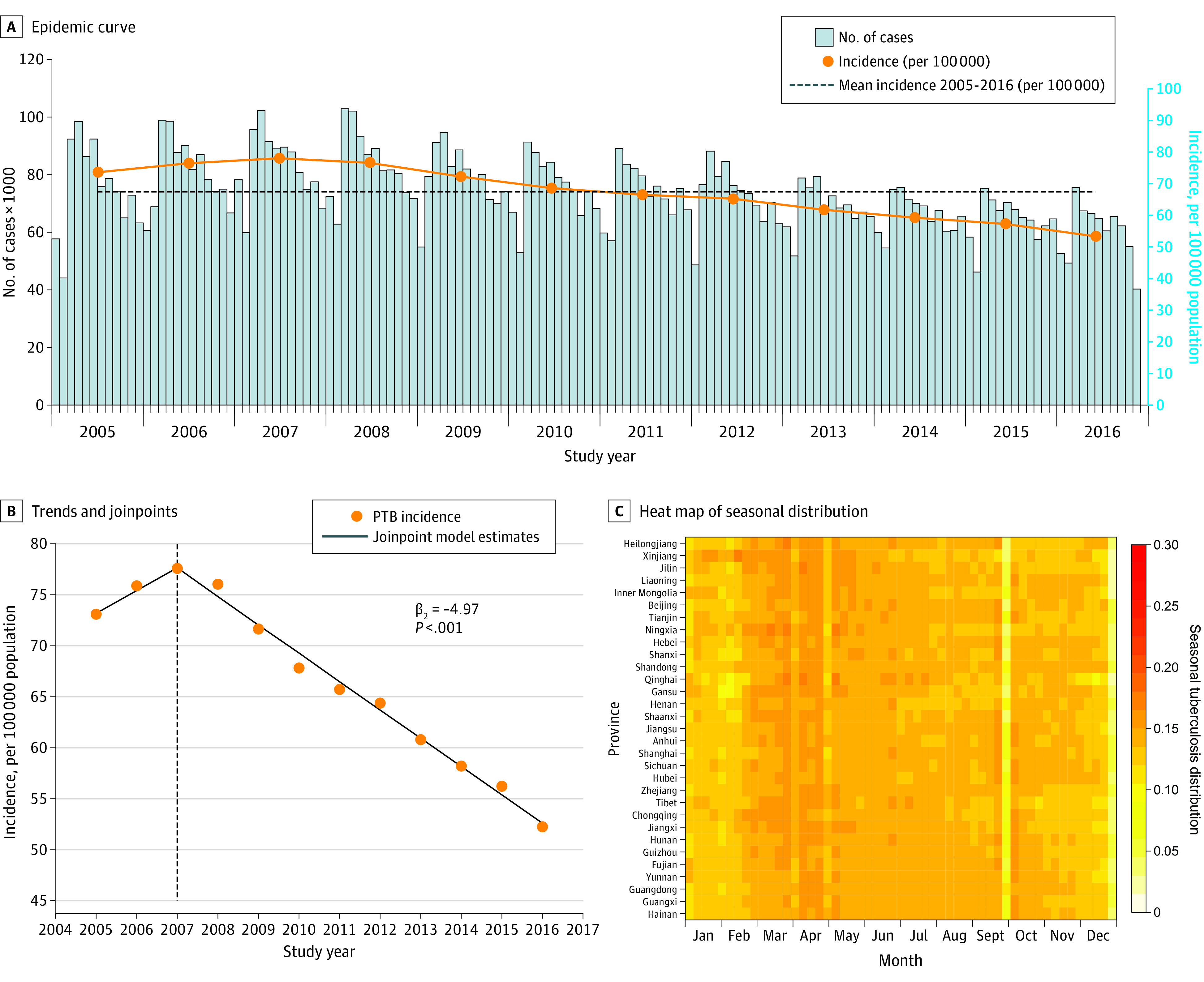
Temporal Pattern and Trends of Pulmonary Tuberculosis (PTB) in Mainland China, January 2005 to November 2016 A, Epidemic curve of PTB cases by year. B, Trends and joinpoints of PTB incidence. For the joinpoint model, PTB incidence = β_0_ + β_1_ (year) + β_2_ (year + 2007) + E. C, Heat map of weekly proportion of PTB cases by province.

The annual incidence of PTB in mainland China initially increased from 72.95 per 100 000 population in 2005 to 77.53 per 100 000 population in 2007, followed by a gradual but steady decline to 52.18 per 100 000 population in 2016 (Figure 1A and B). The 12-year mean annual incidence was 66.61 per 100 000 population. The mean annual incidence declined from 75.45 per 100 000 during 2005 to 2007 to 63.67 per 100 000 during 2008 to 2016. The temporal trend of incidence in the Han ethnic group obtained from the joinpoint regression resembles the national trend, although with 3 rather than 2 slopes (eFigure 2A in the Supplement). For all other ethnic groups, the peak incidence was reached in 2010, and the subsequent decline resulted in a much lower rate (eFigure 2B and eTable 2 in the Supplement).

At the national level, the reporting of PTB exhibits a clear seasonal pattern, jumping from the valley near January or February to the peak in March or April and then declining gradually over the rest of the year (Figure 1C). Spring Festival holidays in February (occasionally in January) could have partially contributed to the lower reporting in the month. The holiday effect is also seen from the slightly higher case numbers in November than in October for most of the years due to the holiday week associated with National Day on October 1.

**Figure 2.**
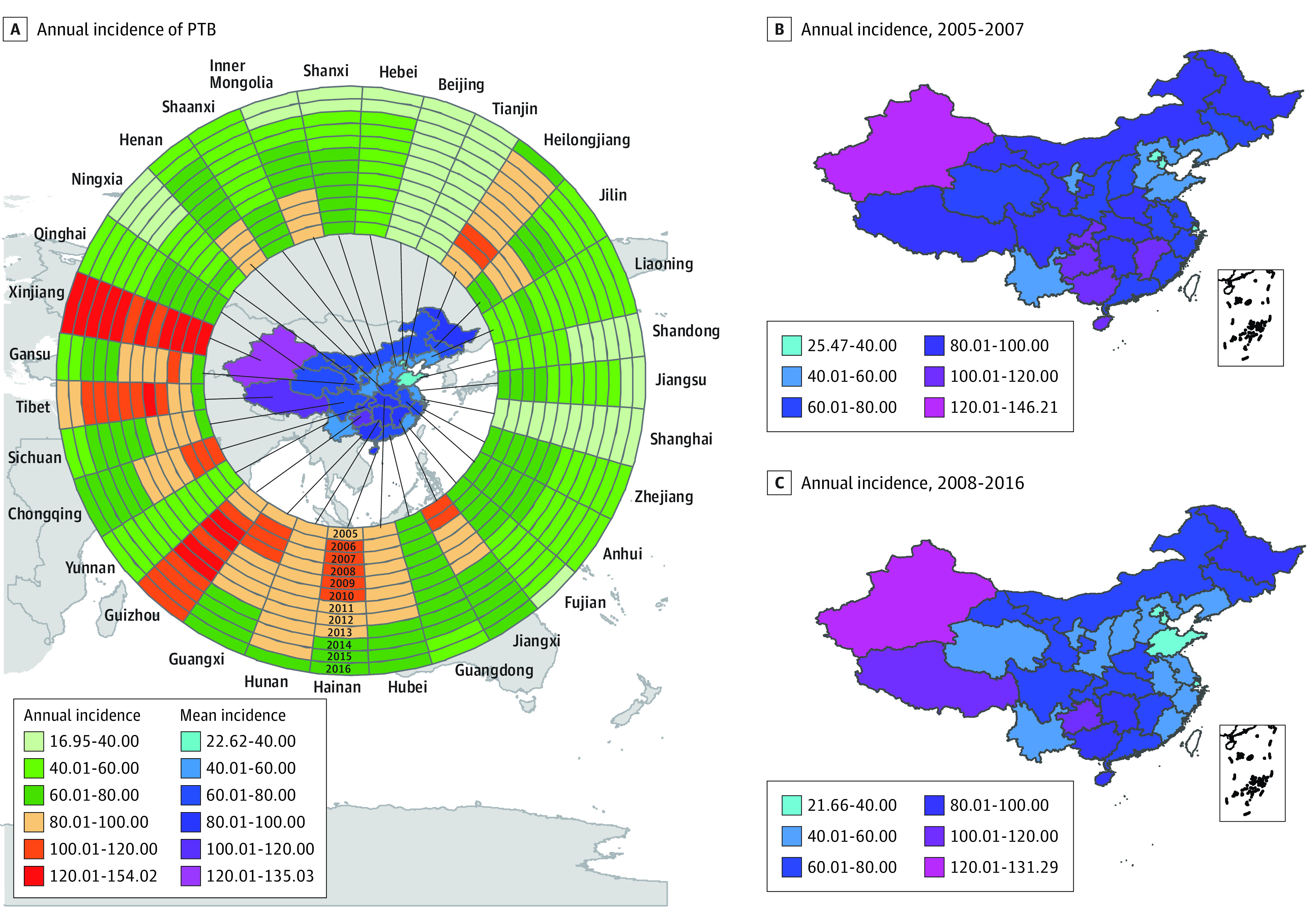
Spatiotemporal Distribution of Patients With Pulmonary Tuberculosis (PTB) in Mainland China, January 2005 to November 2016 A, Annual and mean incidence of PTB per 100 000 population in the 31 Chinese provinces investigated. The 12 rings contain data for each year studied, starting from the innermost ring in 2005 to the outermost in 2016. B, Map of the mean annual incidence per 100 000 population of PTB by region before (2005-2007) the decrease in incidence based on the annual incidence per 100 000 population in mainland China. C, Map of the mean annual incidence of PTB by region after (2008-2016) the decrease in the incidence based on the annual incidence per 100 000 population in mainland China.

Mean PTB incidence was relatively high in the west (Tibet [101.98 per 100 000 population]), northwest (Xinjiang [135.03 per 100 000 population]), central south (Guizhou [115.98 per 100 000 population], Hainan [94.02 per 100 000 population], Guangxi [86.23 per 100 000 population], Chongqing [84.98 per 100 000 population], Jiangxi [84.33 per 100 000 population], Hunan [82.92 per 100 000 population], and Hubei [81.63 per 100 000 population]), and northeast (Heilongjiang [91.39 per 100 000 population]) of mainland China (Figure 2A). Xinjiang had consistently leading incidences over the study period, followed by Guizhou, Tibet, and Heilongjiang. Inner Mongolia, Gansu, Sichuan, Chongqing, Henan, Hubei, Guangxi, and Jiangxi had reduced their annual incidences from greater than 80 per 100 000 population during 2005 to 2007 to less than 80 per 100 000 population during 2008 to 2016 (Figure 2B and C). The greatest reduction in the mean (SD) annual incidence (2008-2016 vs 2005-2007) was observed in Inner Mongolia (difference of 28.70 per 100 000 population), Jiangxi (difference of 28.08 per 100 000 population), and Chongqing (difference of 26.04 per 100 000 population). In contrast, the coastal provinces, together with Qinghai (60.40 [SD, 10.10] per 100 000 population), Shanxi (58.64 [SD, 13.74] per 100 000 population), Shaanxi (57.59 [SD, 7.95] per 100 000 population), Yunnan (50.21 [SD, 1.96] per 100 000 population), and Ningxia (46.75 [SD, 8.82] per 100 000 population) maintained relatively low annual incidences throughout the study period (Figure 2A).

From 2005 to 2016, the national incidence of all patients with PTB declined by 28.5% (from 72.95 to 52.18 per 100 000 population) (Table 2). The reduction of incidence in the western region (21.0%; from 82.06 to 64.82 per 100 000 population) was less than that in the eastern and central regions (31.6%; from 69.43 to 47.48 per 100 000 population). The incidence in pediatric PTB (aged 0-14 years) fell dramatically by 68.1% (from 5.44 to 1.73 per 100 000 population) compared with 31.7% (88.71 to 60.60 per 100 000 population) to 39.5% (from 68.38 to 41.37 per 100 000 population) in older groups. In addition, the group aged 0 to 14 years was the only group with a substantial decline (26.0%; from 5.44 to 4.03 per 100 000 population) from 2005 to 2007 (Table 2). In China, more patients with PTB were diagnosed by chest radiography, especially in the western region, as shown by the annual incidences in 2005 (75.66 per 100 000 population), 2007 (86.81 per 100 000 population), and 2016 (63.80 per 100 000 population) in Table 2. The level of reduction in incidence from 2005 to 2016 also varies by diagnostic approach, more than doubling for culture-positive (61.1%; from 40.97 to 15.94 per 100 000 population) and smear-positive (62.5%; from 40.97 to 15.35 per 100 000 population) PTB compared with PTB with a radiographic abnormality (23.4%; from 67.18 to 51.46 per 100 000 population). The difference in the overall PTB reduction rate between the western region and the eastern and central regions was mainly driven by the between-region difference in the reduction rate of patients with PTB detected on radiography.

**Table 2.** Comparison of Pulmonary Tuberculosis (PTB) Incidence of Different Test Results in Different Regions, Age Groups, and Years

Diagnostic criteria by characteristic	Incidence per 100 000 population			Change in incidence, %^a^		
	2005	2007	2016	2005-2007	2007-2016	2005-2016
All PTB	72.95	77.53	52.18	6.3	−32.7	−28.5
Region						
Eastern and central	69.43	71.76	47.48	3.4	−33.8	−31.6
Western	82.06	92.60	64.82	12.9	−30.0	−21.0
Age group, y						
0-14	5.44	4.03	1.73	−26.0	−57.0	−68.1
15-29	80.51	84.76	54.94	5.3	−35.2	−31.8
30-44	68.38	69.77	41.37	2.0	−40.7	−39.5
45-59	88.71	87.46	60.60	−1.4	−30.7	−31.7
≥60	149.90	155.59	101.39	3.8	−34.8	−32.4
Culture-positive PTB						
Overall	40.97	39.21	15.94	−4.3	−59.4	−61.1
Eastern and central region	40.40	38.20	15.98	−5.4	−58.2	−60.4
Western region	42.47	41.86	15.85	−1.4	−62.1	−62.7
Smear-positive PTB						
Overall	40.97	39.12	15.35	−4.5	−60.8	−62.5
Eastern and central region	40.39	38.12	15.31	−5.6	−59.9	−62.1
Western region	42.47	41.74	15.47	−1.7	−62.9	−63.6
Abnormal radiography finding						
Overall	67.18	73.44	51.46	9.3	−29.9	−23.4
Eastern and central region	63.89	68.33	46.88	6.9	−31.4	−26.6
Western region	75.66	86.81	63.80	14.8	−26.5	−15.7

^a^ Incidence change was defined as the incidence in the previous year minus that in the first year, then divided by the first year’s incidence and multiplied by 100.

The temporal trend of PTB incidence differs between the Han population and other ethnic minority groups (eFigure 2 in the Supplement). The incidence trajectory of the Han population resembles the national trend (Figure 1B and eFigure 2A in the Supplement). Enormous increases in incidence occurred among the ethnic minority groups in 2008 (62.71 per 100 000 population) to 2009 (110.94 per 100 000 population) (eTable 2 in the Supplement), mainly driven by improved diagnostic standards and campaigns among these populations. An 11.7% decrease (from 115.62 to 102.10 per 100 000 population) in PTB incidence from 2010 to 2016 was observed among the ethnic minority populations.

### Delay in Diagnosis

**Figure 3.**
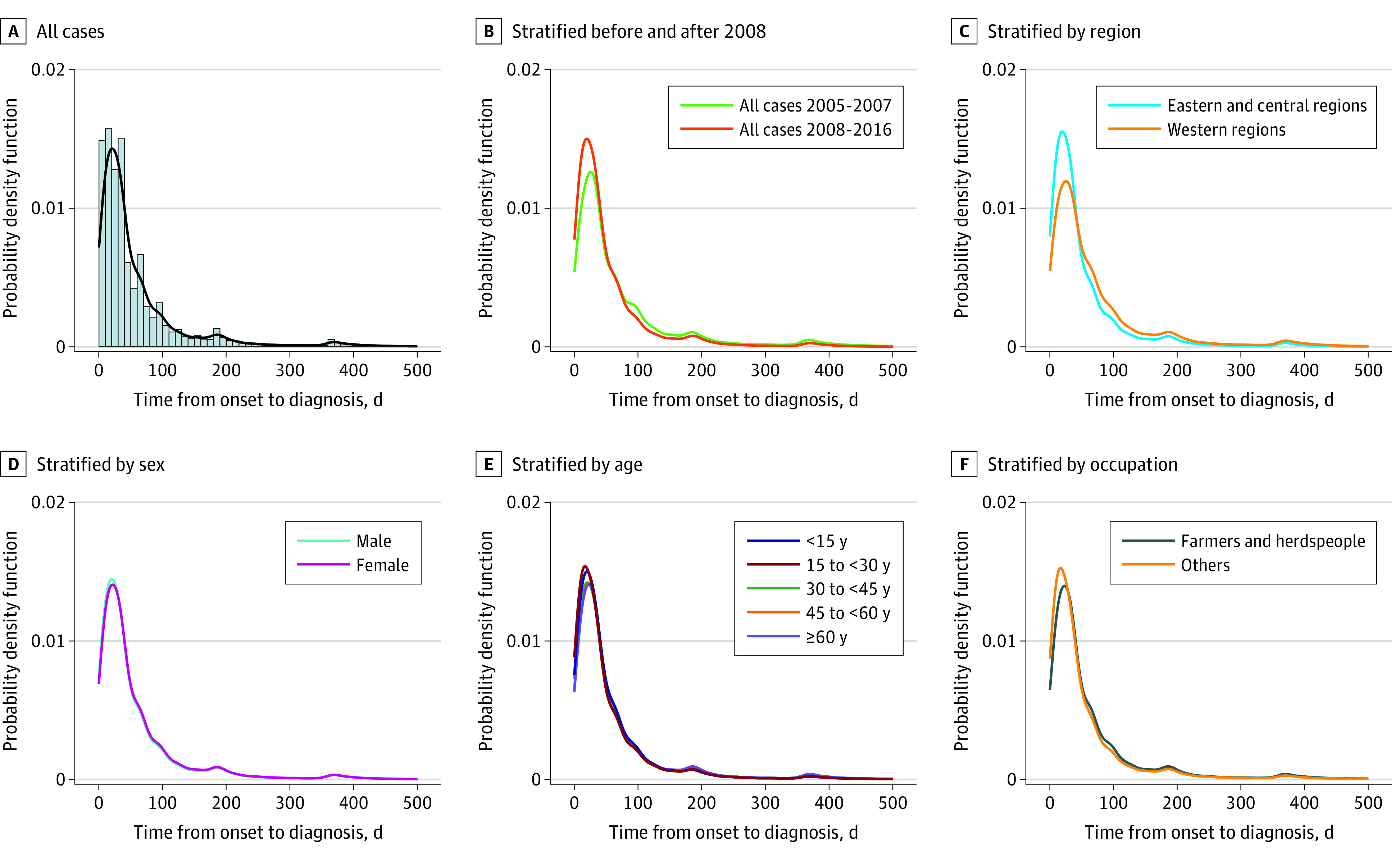
Duration of Time From Illness Onset to Diagnosis of Pulmonary Tuberculosis (PTB) in Mainland China, January 2005 to November 2016 A, For all patients, median duration is 32 (interquartile range [IQR], 15-67) days. B, For patients before 2008, median duration is 36 (IQR, 16-92) days; patients in 2008 and later, 31 (IQR, 15-63) days. C, For patients in the eastern and central regions, median duration is 30 (IQR, 13-61) days; in the western region, 41 (IQR, 20-91) days. D, For male patients, median duration is 32 (IQR, 15-68) days; female, 33 (IQR, 15-69) days. E, For patients younger than 15 years, median duration is 31 (IQR, 14-62) days; aged 30 to 44 years, 32 (IQR, 15-68) days; aged 45 to 59 years, 33 (IQR, 16-70) days; and 60 years or older, 34 (IQR, 16-71) days. F, For patients among farmers and herders, median duration was 33 (IQR, 16-71) days; other occupations, 30 (IQR, 12-61) days.

There was a median delay of 32 (IQR, 15-67) days from disease onset to diagnosis among all patients with reported PTB during the study period (Figure 3A). The median delay was shortened from 36 (IQR, 16-92) days in the period from 2005 to 2007 to 31 (IQR, 15-63) days in the period from 2008 to 2016 (*P* < .001) (Figure 3B). There was also a clear geographic difference, with a median of 30 (IQR, 13-61) days in the eastern and central regions vs 41 (IQR, 20-91) days in the western region (*P* < .001) (Figure 3C). We did not find a difference in median delay for male vs female individuals (32 [IQR, 15-68] vs 33 [IQR, 15-69] days) (Figure 3D). Pediatric and adult patients had similar delay times (Figure 3E), but the delay tended to be longer among the group 60 years or older (median, 34 [IQR, 16-71] days] compared with the group younger than 15 years (median, 31 [IQR, 14-62] days). Farmers and herders (median, 33 [IQR, 16-71] days]) had a slightly longer diagnosis delay than other occupational groups (median, 30 [IQR, 12-61] days), with *P* < .001 (Figure 3F).

## Discussion

We summarized epidemiological characteristics of more than 10 million patients with PTB reported from 2005 to 2016 in mainland China, which is, to our knowledge, the largest epidemiological study of TB in the country. We provided a thorough descriptive analysis of the temporal trend of PTB incidence at the national level as well as by demographic and geographic subpopulations.

According to a report by the World Health Organization, the global incidence rate of TB cases has shown a steady decrease since 2000.^4,14^ We have shown a decline of more than 28% in all patients with PTB from 2007 to 2016. The incidence of culture-positive or smear-positive PTB fell by more than 60%. These decreases are most likely attributable to several mass public health interventions. First, China has been expanding the DOTS program in response to the World Health Organization’s Stop TB Strategy. The DOTS program was fully implemented in China starting in 2005, expanding target patients for diagnosis and treatment from those with positive and negative smear findings to all patients with PTB. Second, the Chinese government revised a law pertaining to the control of infectious diseases in 2004, which mandated reporting of patients with new or relapsed TB to local public health authorities via an internet-based reporting system within 24 hours.^15^ In addition, the Ministry of Health issued a policy to strengthen collaboration between the hospitals and TB dispensaries on diagnosis and treatment, which doubled the TB discovery rate.^15^ Last, free diagnostic testing and therapies for patients with TB were made available nationwide starting in 2004, which greatly reduced the economic burden of patients, shortened the diagnosis delay, and improved treatment adherence and outcomes.^16,17^

Although the incidence of TB has been declining in China since 2007, the targets of a 90% reduction in incidence and a 95% reduction in mortality by 2035 remain difficult to achieve, even if all existing interventions are scaled up.^5^ It is therefore crucial to target effective interventions at high-risk populations and areas that have been underserved. Our study found that male farmers and herdsmen, especially those in the west, constituted the underserved high-risk subpopulation in China, likely due to tobacco use, corticosteroid use, immunity levels, migration, and living environment.^18^ Tuberculosis-related education programs and community-based TB screening strategies should be tailored to this subpopulation.^5,19^

Compared with the eastern and central regions, the western region has a much lower population density (53.74/km^2^ vs 310.09/ km^2^ to 413.05/km^2^),^12,20^ yet its incidence was higher and declining at a slower rate. A major contributing factor is the less developed socioeconomic infrastructure in the west.^21^ Tuberculosis is known to be a disease associated with poverty.^4^ The gross domestic product per capita during 2005 to 2016 in the vast western region was ¥21.95 thousand compared with ¥47.10 thousand in the eastern and ¥26.22 thousand in the central regions.^22^ Although a low population density helps deter transmission of infectious diseases, it also increases the difficulty of health services reaching people in need. More cost-effective prevention and control strategies, possibly aided by mobile phone technologies, such as TB-dedicated self-screening and compliance-monitoring apps, should be designed for the western region.^5,23^

In addition to economic and logistic factors, social and cultural barriers could explain the higher incidences and slower decline in western and southwestern provinces such as Xinjiang (135.03 per 100 000 population), Guizhou Province (115.98 per 100 000 population), and Tibet (101.98 per 100 000 population), where many ethnic minority groups reside. The substantial rise of incidence from 2005 to 2010 among ethnic minority groups suggests severe underdetection of TB cases rather than increasing transmission (which is unlikely in such a short time frame) in these populations. The slow decline of incidence further suggests that the existing prevention and control strategies might not have reached their full effectiveness in these populations. Educational and interventional programs may need further improvement to be culturally well accepted. In addition, diagnosis and treatment plans should consider potential genetic diversity of *M tuberculosis* (eg, the dominant strain in the western region is *M tuberculosis* lineage 4).^24^

Delay in diagnosis of TB is common in low- and middle-income countries^25^ despite the key role of early diagnosis for effective TB treatment and desired outcomes.^26,27^ Our study showed shortened diagnosis delay from 2005 to 2016 in China. More timely diagnosis could have helped reduce the incidence. On the other hand, longer delay in the western region and among ethnic minority groups could have contributed to their higher incidence and slower decline. As in many other countries, health care resources are much less accessible in rural areas than in urban areas of China, especially in the western region. In 2016, the numbers of hospital beds and medical staff per 1000 population in rural regions were less than half of those in urban areas.^28^ Because accessibility of health care heavily depends on economic development,^29^ the government should encourage investment to develop ecological economies in rural areas.

### Limitations

Our study had several limitations. First, this study only included registered patients with PTB in mainland China, excluding extrapulmonary tuberculosis. However, our description of the overall epidemic situation of TB in China is not affected because more than 90% of patients with TB had PTB. Second, the World Health Organization estimated 13.26 million cases of TB from 2005 to 2016 in mainland China,^30,31,32,33,34,35,36,37,38,39,40,41^ but the TBIMS reported 10.58 million, indicating potential underreporting of TB by the existing surveillance system. Underreporting of TB cases more likely occurred among ethnic minority groups. In addition, we likely overestimated the incidence in 2016 by imputing the December incidence with the mean value in the previous 11 months of the year. Last, the impact of a variety of TB-related public health policies during the study period was not explicitly evaluated in our descriptive analysis, which could be subject to future investigation.

## Conclusions

This cross-sectional study found that, from 2005 to 2016, the incidence of PTB in mainland China showed a downward trend. Comprehensive, scalable, and cost-effective interventions should target high-risk populations, particularly those in rural areas and among ethnic minority groups, to sustain or accelerate the decline toward achieving the World Health Organization’s goal of eliminating TB by 2035.

## Notes

Supplement. **eTable 1.** The Number of Pulmonary Tuberculosis (PTB) Cases in Different Nationalities in Mainland China, 2005 to November 2016 **eTable 2.** The Incidence Changes of Pulmonary Tuberculosis (PTB) in Different Ethnics in Mainland China, 2005 to November 2016 **eFigure 1.** The Age Distribution of Pulmonary Tuberculosis (PTB) Cases by Time and Sex in Mainland China, 2005 to November 2016 **eFigure 2.** Trends and Joinpoints of Incidence of Pulmonary Tuberculosis (PTB) in Han and Other Ethnics Minority Groups, 2005 to November 2016
