# Supplementary material for: Changes in Incidence and Epidemiological Characteristics of Pulmonary Tuberculosis in Mainland China, 2005-2016
Source: JAMA Netw Open. 2021 Apr 9;4(4):e215302. doi: 10.1001/jamanetworkopen.2021.5302 (PMC8035653; doi:10.1001/jamanetworkopen.2021.5302)
Supplement: Supplement. — eTable 1. The Number of Pulmonary Tuberculosis (PTB) Cases in Different Nationalities in Mainland China, 2005 to November 2016 eTable 2. The Incidence Changes of Pulmonary Tuberculosis (PTB) in Different Ethnics in Mainland China, 2005 to November 2016 eFigure 1. The Age Distribution of Pulmonary Tuberculosis (PTB) Cases by Time and Sex in Mainland China, 2005 to November 2016 eFigure 2. Trends and Joinpoints of Incidence of Pulmonary Tuberculosis (PTB) in Han and Other Ethnics Minority Groups, 2005 to November 2016 [file jamanetwopen-e215302-s001.pdf]

Supplementary Online Content

Jiang H, Liu M, Zhang Y, et al. Changes in incidence and epidemiological characteristics of pulmonary tuberculosis in mainland China, 2005-2016. *JAMA Netw Open*. 2021;4(4):e215302.
doi:10.1001/jamanetworkopen.2021.5302

eTable 1. The Number of Pulmonary Tuberculosis (PTB) Cases in Different Nationalities in Mainland China, 2005 to November 2016
eTable 2. The Incidence Changes of Pulmonary Tuberculosis (PTB) in Different Ethnics in Mainland China, 2005 to November 2016
eFigure 1. The Age Distribution of Pulmonary Tuberculosis (PTB) Cases by Time and Sex in Mainland China, 2005 to November 2016
eFigure 2. Trends and Joinpoints of Incidence of Pulmonary Tuberculosis (PTB) in Han and Other Ethnics Minority Groups, 2005 to November 2016

This supplementary material has been provided by the authors to give readers additional information about their work.

© 2021 Jiang H et al. *JAMA Network Open*.

eTable 1. The Number of Pulmonary Tuberculosis (PTB) Cases in Different Nationalities in Mainland China, 2005 to November 2016

Nationality	2005	2006	2007	2008	2009	2010	2011	2012	2013	2014	2015	2016	total
Han	937,108 (99.88)	981,465 (99.87)	1,008,051 (99.72)	945,358 (94.59)	851,638 (89.81)	803,706 (88.75)	781,514 (88.75)	765,384 (88.16)	723,675 (87.81)	695,285 (87.55)	671,046 (87.08)	569,652 (86.31)	9,733,882 (91.98)
Uighur	52 (0.01)	169 (0.02)	1,005 (0.10)	22,836 (2.29)	17,678 (1.86)	19,223 (2.12)	17,138 (1.95)	21,515 (2.48)	23,355 (2.83)	23,582 (2.97)	25,597 (3.32)	23,858 (3.61)	196,008 (1.85)
Zhuang	29 (0.00)	65 (0.01)	454 (0.04)	10,991 (1.10)	15,736 (1.66)	16,779 (1.85)	15,442 (1.75)	14,997 (1.73)	14,319 (1.74)	13,681 (1.72)	13,431 (1.74)	11,995 (1.82)	127,919 (1.21)
Tujiav	269 (0.03)	449 (0.05)	461 (0.05)	1,907 (0.19)	9,235 (0.97)	9,258 (1.02)	9,277 (1.05)	9,783 (1.13)	8,477 (1.03)	8,559 (1.08)	8,826 (1.15)	7,184 (1.09)	73,685 (0.70)
Miao	269 (0.03)	264 (0.03)	190 (0.02)	2,129 (0.21)	8,155 (0.86)	8,295 (0.92)	8,959 (1.02)	8,794 (1.01)	8,304 (1.01)	8,061 (1.02)	8,170 (1.06)	7,399 (1.12)	68,989 (0.65)

© 2021 Jiang H et al. *JAMA Network Open*.

Yi	21 (0.00)	29 (0.00)	122 (0.01)	2,229 (0.22)	6,433 (0.68)	7,189 (0.79)	7,723 (0.88)	7,703 (0.89)	7,639 (0.93)	7,424 (0.93)	7,318 (0.95)	7,396 (1.12)	61,226 (0.58)
Tibetan	103 (0.01)	62 (0.01)	192 (0.02)	4,666 (0.47)	7,592 (0.80)	7,358 (0.81)	7,358 (0.84)	7,105 (0.82)	7,061 (0.86)	6,842 (0.86)	6,388 (0.83)	5,889 (0.89)	60,616 (0.57)
Hui	62 (0.01)	15 (0.00)	14 (0.00)	1,768 (0.18)	4,368 (0.46)	4,617 (0.51)	4,260 (0.48)	4,076 (0.47)	4,215 (0.51)	4,174 (0.53)	3,750 (0.49)	3,350 (0.51)	34,669 (0.33)
Mongolia	81 (0.01)	84 (0.01)	88 (0.01)	1,266 (0.13)	4,586 (0.48)	4,586 (0.51)	4,464 (0.51)	4,365 (0.50)	4,171 (0.51)	3,684 (0.46)	3,613 (0.47)	3,137 (0.48)	34,125 (0.32)
Buyi	0 (0.00)	0 (0.00)	1 (0.00)	331 (0.03)	3,106 (0.33)	3,718 (0.41)	3,676 (0.42)	3,656 (0.42)	3,253 (0.39)	3,264 (0.41)	3,479 (0.45)	3,097 (0.47)	27,581 (0.26)
Manchu	32 (0.00)	0 (0.00)	38 (0.00)	78 (0.01)	3,278 (0.35)	3,697 (0.41)	3,397 (0.39)	3,172 (0.37)	2,886 (0.35)	2,583 (0.33)	2,430 (0.32)	2,245 (0.34)	23,836 (0.23)
Dong	0 (0.00)	0 (0.00)	11 (0.00)	1,272 (0.13)	3,076 (0.32)	2,914 (0.32)	3,077 (0.35)	2,954 (0.34)	2,615 (0.32)	2,462 (0.31)	2,280 (0.30)	2,324 (0.35)	22,985 (0.22)

© 2021 Jiang H et al. *JAMA Network Open*.

Hasa	13<br>(0.00)	22<br>(0.00)	41<br>(0.00)	1,821<br>(0.18)	1,878<br>(0.20)	2,009<br>(0.22)	1,916<br>(0.22)	2,120<br>(0.24)	2,113<br>(0.26)	2,175<br>(0.27)	2,075<br>(0.27)	1,792<br>(0.27)	17,975<br>(0.17)
Li	0<br>(0.00)	0<br>(0.00)	1<br>(0.00)	877<br>(0.09)	1,664<br>(0.18)	1,668<br>(0.18)	1,671<br>(0.19)	1,613<br>(0.19)	1,536<br>(0.19)	1,550<br>(0.20)	1,611<br>(0.21)	1,507<br>(0.23)	13,698<br>(0.13)
Yao	0<br>(0.00)	0<br>(0.00)	4<br>(0.00)	301<br>(0.03)	1,552<br>(0.16)	1,694<br>(0.19)	1,782<br>(0.20)	1,781<br>(0.21)	1,661<br>(0.20)	1,685<br>(0.21)	1,683<br>(0.22)	1,491<br>(0.23)	13,634<br>(0.13)
Hani	90<br>(0.01)	75<br>(0.01)	69<br>(0.01)	391<br>(0.04)	1,397<br>(0.15)	1,554<br>(0.17)	1,600<br>(0.18)	1,782<br>(0.21)	1,688<br>(0.20)	1,625<br>(0.20)	1,679<br>(0.22)	1,398<br>(0.21)	13,348<br>(0.13)
Korea	1<br>(0.00)	0<br>(0.00)	2<br>(0.00)	228<br>(0.02)	1,467<br>(0.15)	1,504<br>(0.17)	1,351<br>(0.15)	1,233<br>(0.14)	973<br>(0.12)	817<br>(0.10)	751<br>(0.10)	622<br>(0.09)	8,949<br>(0.08)
Bai	7<br>(0.00)	4<br>(0.00)	5<br>(0.00)	34<br>(0.00)	1,100<br>(0.12)	1,095<br>(0.12)	1,022<br>(0.12)	996<br>(0.11)	988<br>(0.12)	1,022<br>(0.13)	1,050<br>(0.14)	936<br>(0.14)	8,259<br>(0.08)
Lisu	0<br>(0.00)	0<br>(0.00)	0<br>(0.00)	47<br>(0.00)	624<br>(0.07)	685<br>(0.08)	711<br>(0.08)	693<br>(0.08)	673<br>(0.08)	691<br>(0.09)	663<br>(0.09)	562<br>(0.09)	5,349<br>(0.05)

© 2021 Jiang H et al. *JAMA Network Open*.

Dai	26<br>(0.00)	17<br>(0.00)	34<br>(0.00)	138<br>(0.01)	372<br>(0.04)	374<br>(0.04)	472<br>(0.05)	498<br>(0.06)	450<br>(0.05)	511<br>(0.06)	464<br>(0.06)	541<br>(0.08)	3,897<br>(0.04)
Dongxiang	0<br>(0.00)	1<br>(0.00)	0<br>(0.00)	92<br>(0.01)	514<br>(0.05)	516<br>(0.06)	485<br>(0.06)	467<br>(0.05)	444<br>(0.05)	433<br>(0.05)	444<br>(0.06)	295<br>(0.04)	3,691<br>(0.03)
Va	8<br>(0.00)	9<br>(0.00)	9<br>(0.00)	181<br>(0.02)	318<br>(0.03)	426<br>(0.05)	476<br>(0.05)	442<br>(0.05)	465<br>(0.06)	444<br>(0.06)	435<br>(0.06)	390<br>(0.06)	3,603<br>(0.03)
Lahu	16<br>(0.00)	11<br>(0.00)	13<br>(0.00)	32<br>(0.00)	361<br>(0.04)	414<br>(0.05)	468<br>(0.05)	427<br>(0.05)	321<br>(0.04)	328<br>(0.04)	282<br>(0.04)	281<br>(0.04)	2,954<br>(0.03)
Gelao	0<br>(0.00)	0<br>(0.00)	0<br>(0.00)	2<br>(0.00)	257<br>(0.03)	226<br>(0.02)	177<br>(0.02)	311<br>(0.04)	251<br>(0.03)	363<br>(0.05)	578<br>(0.08)	460<br>(0.07)	2,625<br>(0.02)
Kirghiz	0<br>(0.00)	0<br>(0.00)	0<br>(0.00)	123<br>(0.01)	216<br>(0.02)	291<br>(0.03)	222<br>(0.03)	299<br>(0.03)	263<br>(0.03)	252<br>(0.03)	296<br>(0.04)	280<br>(0.04)	2,242<br>(0.02)
Shui	0<br>(0.00)	0<br>(0.00)	1<br>(0.00)	10<br>(0.00)	255<br>(0.03)	221<br>(0.02)	290<br>(0.03)	257<br>(0.03)	234<br>(0.03)	264<br>(0.03)	268<br>(0.03)	262<br>(0.04)	2,062<br>(0.02)

© 2021 Jiang H et al. *JAMA Network Open*.

She	0 (0.00)	0 (0.00)	1 (0.00)	10 (0.00)	266 (0.03)	279 (0.03)	220 (0.02)	188 (0.02)	206 (0.02)	194 (0.02)	182 (0.02)	171 (0.03)	1,717 (0.02)
Qiang	0 (0.00)	0 (0.00)	31 (0.00)	41 (0.00)	188 (0.02)	179 (0.02)	192 (0.02)	151 (0.02)	150 (0.02)	150 (0.02)	120 (0.02)	97 (0.01)	1,299 (0.01)
Mulam	0 (0.00)	0 (0.00)	0 (0.00)	8 (0.00)	109 (0.01)	137 (0.02)	164 (0.02)	148 (0.02)	151 (0.02)	154 (0.02)	145 (0.02)	141 (0.02)	1,157 (0.01)
Daur	0 (0.00)	0 (0.00)	0 (0.00)	21 (0.00)	139 (0.01)	130 (0.01)	125 (0.01)	99 (0.01)	129 (0.02)	81 (0.01)	68 (0.01)	78 (0.01)	870 (0.01)
Naxi	36 (0.00)	44 (0.00)	56 (0.01)	56 (0.01)	105 (0.01)	81 (0.01)	80 (0.01)	95 (0.01)	90 (0.01)	76 (0.01)	88 (0.01)	58 (0.01)	865 (0.01)
Du	1 (0.00)	1 (0.00)	0 (0.00)	1 (0.00)	107 (0.01)	116 (0.01)	119 (0.01)	112 (0.01)	126 (0.02)	96 (0.01)	86 (0.01)	80 (0.01)	845 (0.01)
Jingpo	0 (0.00)	0 (0.00)	2 (0.00)	27 (0.00)	69 (0.01)	79 (0.01)	107 (0.01)	113 (0.01)	100 (0.01)	108 (0.01)	98 (0.01)	98 (0.01)	801 (0.01)

© 2021 Jiang H et al. *JAMA Network Open*.

Maonan	0 (0.00)	0 (0.00)	0 (0.00)	8 (0.00)	69 (0.01)	65 (0.01)	53 (0.01)	30 (0.00)	31 (0.00)	32 (0.00)	42 (0.01)	36 (0.01)	366 (0.00)
Blang	0 (0.00)	0 (0.00)	0 (0.00)	2 (0.00)	34 (0.00)	50 (0.01)	28 (0.00)	43 (0.00)	42 (0.01)	40 (0.01)	47 (0.01)	66 (0.01)	352 (0.00)
Salar	0 (0.00)	0 (0.00)	0 (0.00)	35 (0.00)	49 (0.01)	47 (0.01)	44 (0.00)	39 (0.00)	41 (0.00)	31 (0.00)	29 (0.00)	13 (0.00)	328 (0.00)
Xibe	0 (0.00)	0 (0.00)	0 (0.00)	9 (0.00)	25 (0.00)	31 (0.00)	32 (0.00)	35 (0.00)	67 (0.01)	31 (0.00)	39 (0.01)	33 (0.00)	302 (0.00)
Evenki	0 (0.00)	0 (0.00)	0 (0.00)	32 (0.00)	43 (0.00)	46 (0.01)	37 (0.00)	30 (0.00)	29 (0.00)	38 (0.00)	25 (0.00)	21 (0.00)	301 (0.00)
Tajik	0 (0.00)	0 (0.00)	0 (0.00)	0 (0.00)	41 (0.00)	41 (0.00)	39 (0.00)	29 (0.00)	33 (0.00)	25 (0.00)	47 (0.01)	41 (0.01)	296 (0.00)
Nu	0 (0.00)	0 (0.00)	0 (0.00)	2 (0.00)	18 (0.00)	27 (0.00)	30 (0.00)	38 (0.00)	44 (0.01)	32 (0.00)	45 (0.01)	22 (0.00)	258 (0.00)
Jino	2 (0.00)	0 (0.00)	0 (0.00)	12 (0.00)	19 (0.00)	14 (0.00)	19 (0.00)	22 (0.00)	16 (0.00)	26 (0.00)	30 (0.00)	39 (0.01)	199 (0.00)
Pumi	0 (0.00)	0 (0.00)	0 (0.00)	0 (0.00)	17 (0.00)	30 (0.00)	13 (0.00)	23 (0.00)	16 (0.00)	34 (0.00)	36 (0.00)	21 (0.00)	190 (0.00)
Menba	0 (0.00)	0 (0.00)	0 (0.00)	1 (0.00)	7 (0.00)	8 (0.00)	27 (0.00)	28 (0.00)	25 (0.00)	18 (0.00)	18 (0.00)	14 (0.00)	146 (0.00)
Gin	0 (0.00)	0 (0.00)	0 (0.00)	1 (0.00)	18 (0.00)	13 (0.00)	13 (0.00)	19 (0.00)	20 (0.00)	12 (0.00)	20 (0.00)	17 (0.00)	133 (0.00)
Yugur	0 (0.00)	0 (0.00)	0 (0.00)	0 (0.00)	16 (0.00)	13 (0.00)	25 (0.00)	15 (0.00)	14 (0.00)	22 (0.00)	13 (0.00)	14 (0.00)	132 (0.00)
Oroqin	0 (0.00)	0 (0.00)	0 (0.00)	3 (0.00)	15 (0.00)	16 (0.00)	11 (0.00)	9 (0.00)	17 (0.00)	10 (0.00)	9 (0.00)	6 (0.00)	96 (0.00)
Bonan	0 (0.00)	0 (0.00)	0 (0.00)	0 (0.00)	14 (0.00)	22 (0.00)	8 (0.00)	10 (0.00)	17 (0.00)	14 (0.00)	4 (0.00)	3 (0.00)	92 (0.00)

© 2021 Jiang H et al. *JAMA Network Open*.

De'ang	0 (0.00)	0 (0.00)	0 (0.00)	3 (0.00)	13 (0.00)	5 (0.00)	6 (0.00)	14 (0.00)	13 (0.00)	8 (0.00)	7 (0.00)	9 (0.00)	78 (0.00)
Achang	0 (0.00)	0 (0.00)	0 (0.00)	1 (0.00)	6 (0.00)	8 (0.00)	14 (0.00)	12 (0.00)	6 (0.00)	10 (0.00)	6 (0.00)	3 (0.00)	66 (0.00)
Lhoba	0 (0.00)	0 (0.00)	0 (0.00)	0 (0.00)	0 (0.00)	0 (0.00)	13 (0.00)	7 (0.00)	13 (0.00)	10 (0.00)	6 (0.00)	15 (0.00)	64 (0.00)
Drung	0 (0.00)	0 (0.00)	1 (0.00)	0 (0.00)	4 (0.00)	7 (0.00)	13 (0.00)	8 (0.00)	8 (0.00)	9 (0.00)	6 (0.00)	7 (0.00)	63 (0.00)
Russian	0 (0.00)	0 (0.00)	0 (0.00)	1 (0.00)	2 (0.00)	6 (0.00)	5 (0.00)	2 (0.00)	4 (0.00)	6 (0.00)	1 (0.00)	1 (0.00)	28 (0.00)
Uzbek	0 (0.00)	0 (0.00)	0 (0.00)	1 (0.00)	2 (0.00)	3 (0.00)	3 (0.00)	2 (0.00)	2 (0.00)	3 (0.00)	4 (0.00)	3 (0.00)	23 (0.00)
Gaoshan	0 (0.00)	0 (0.00)	0 (0.00)	0 (0.00)	1 (0.00)	0 (0.00)	1 (0.00)	3 (0.00)	4 (0.00)	3 (0.00)	2 (0.00)	2 (0.00)	16 (0.00)
Tatar	0 (0.00)	0 (0.00)	0 (0.00)	1 (0.00)	1 (0.00)	2 (0.00)	2 (0.00)	2 (0.00)	1 (0.00)	1 (0.00)	0 (0.00)	3 (0.00)	13 (0.00)
Hezhen	0 (0.00)	0 (0.00)	0 (0.00)	0 (0.00)	1 (0.00)	0 (0.00)	0 (0.00)	1 (0.00)	2 (0.00)	1 (0.00)	0 (0.00)	1 (0.00)	6 (0.00)
Unknown	0 (0.00)	0 (0.00)	0 (0.00)	2 (0.00)	56 (0.01)	135 (0.01)	162 (0.02)	426 (0.05)	728 (0.09)	1,134 (0.14)	792 (0.10)	559 (0.08)	3,994 (0.04)
total	938,226 (100.00)	982,786 (100.00)	1,010,897 (100.00)	999,386 (100.00)	948,310 (100.00)	905,607 (100.00)	880,533 (100.00)	868,176 (100.00)	824,175 (100.00)	794,170 (100.00)	770,622 (100.00)	660,015 (100.00)	10,582,903 (100.00)

Data are presented as n (%) of patients unless otherwise indicated.

© 2021 Jiang H et al. *JAMA Network Open*.

eTable 2. The Incidence Changes of Pulmonary Tuberculosis (PTB) in Different Ethnics in Mainland China, 2005 to November 2016

Ethnic	Incidence per 10, 000 population												Change* (%)		
	2005	2006	2007	2008	2009	2010	2011	2012	2013	2014	2015	2016	2005-2007§/ 2005-2010£	2007-2016/ 2010-2016	2005-2016
Han	77.86	81.01	82.70	76.97	68.83	64.52	62.46	60.88	57.32	54.67	52.58	48.43	6.2	-41.4	-37.8
Other	1.36	1.58	3.35	62.71	110.94	115.62	111.08	113.38	108.36	109.15	105.29	102.10	8420.2	-11.7	7407.4

*We calculated the incidence changes on the basis of the ethnic. Specifically, the incidence change (%) was defined as the incidence in the previous year minus that in the first year then divided by the first year's incidence and multiplied by 100.

§ Joinpoint regression model showed that the turning point of the incidence of Han ethnic was 2007, so the time point of change is bounded by 2007.

£Joinpoint regression model showed that the turning point of the incidence of Han ethnic was 2010, so the time point of change is bounded by 2010.

© 2021 Jiang H et al. *JAMA Network Open*.

eFigure 1. The Age Distribution of Pulmonary Tuberculosis (PTB) Cases by Time and Sex in Mainland China, 2005 to November 2016

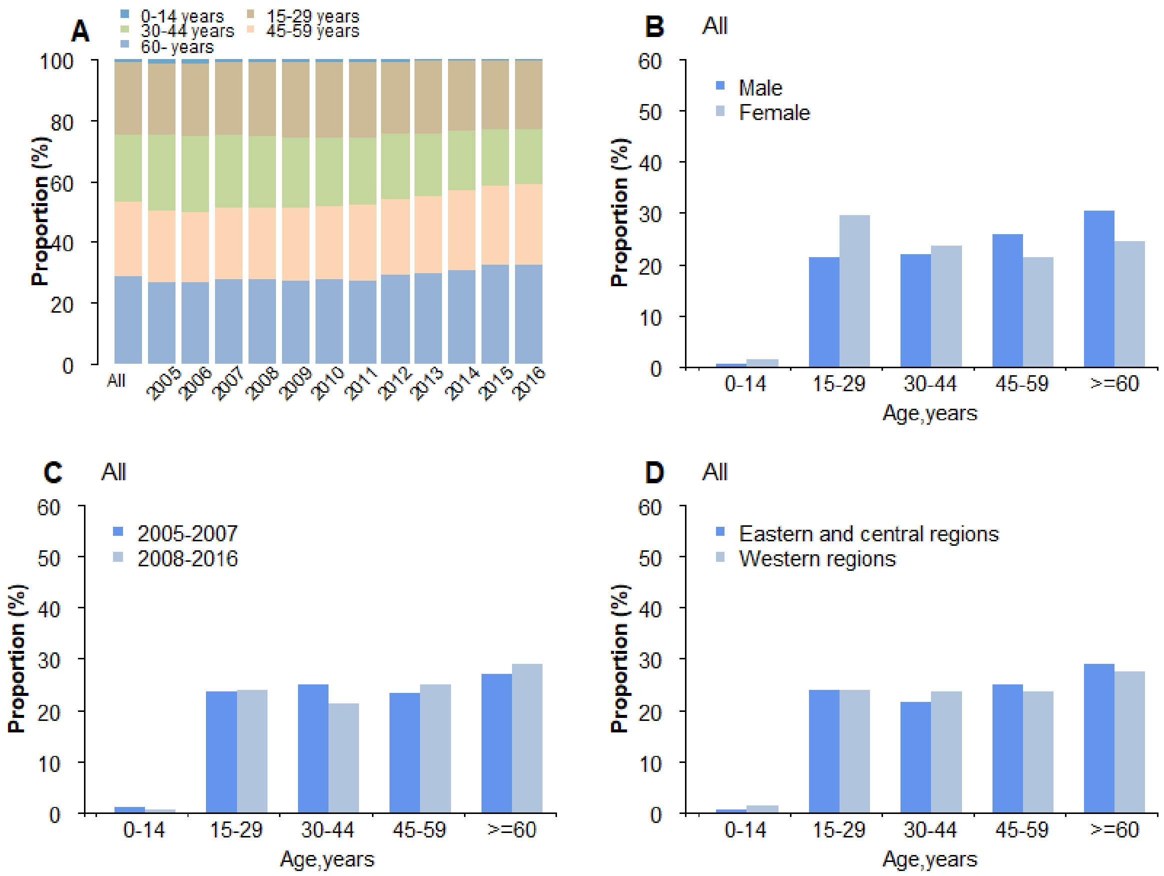

(A) The age distribution of PTB cases by year. (B) The age distribution of PTB cases by sex. (C) The age distribution of PTB cases before and after 1 January 2008. (D) The age distribution of PTB cases in the eastern and central regions and western region.

S2 Table 1. The number of pulmonary tuberculosis (PTB) cases in different nationalities in mainland China, 2005-November 2016.

© 2021 Jiang H et al. *JAMA Network Open*.

eFigure 2. Trends and Joinpoints of Incidence of Pulmonary Tuberculosis (PTB) in Han and Other Ethnics Minority Groups, 2005 to November 2016

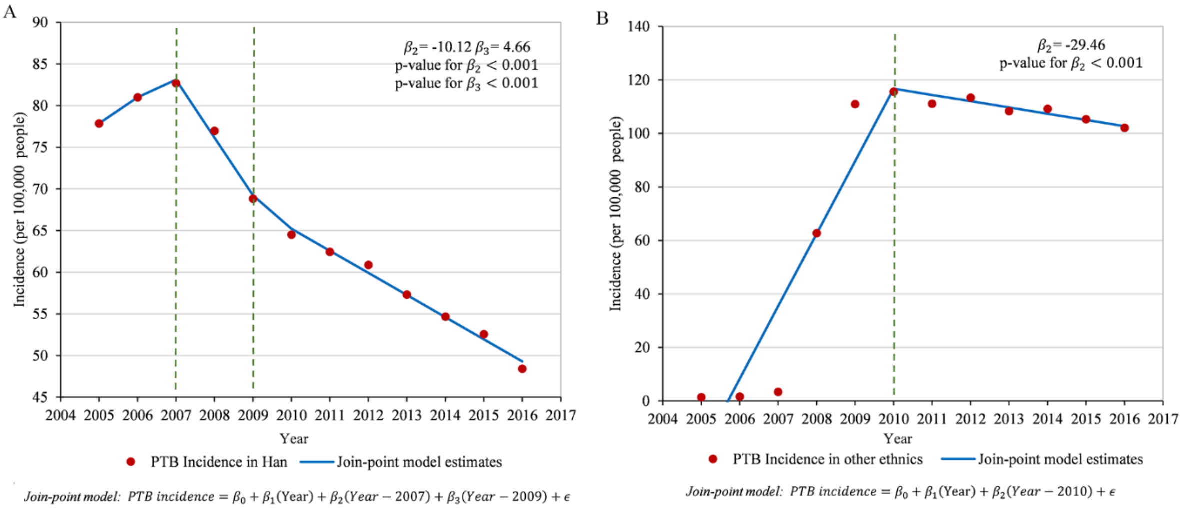

(A) Trends and joinpoints of incidence of pulmonary tuberculosis (PTB) in Han ethnic. (B) Trends and joinpoints of incidence of pulmonary tuberculosis (PTB) in other ethnics minorities

© 2021 Jiang H et al. *JAMA Network Open*.
